# Autoimmunity in Chronic Chagas Disease: A Road of Multiple Pathways to Cardiomyopathy?

**DOI:** 10.3389/fimmu.2018.01842

**Published:** 2018-08-06

**Authors:** Elidiana De Bona, Kárita Cláudia Freitas Lidani, Lorena Bavia, Zahra Omidian, Luiza Helena Gremski, Thaisa Lucas Sandri, Iara J. de Messias Reason

**Affiliations:** ^1^Laboratory of Molecular Immunopathology, Department of Clinical Pathology, Federal University of Paraná, Curitiba, Brazil; ^2^Department of Pathology, Division of Immunology, School of Medicine, Johns Hopkins University, Baltimore, MD, United States; ^3^Department of Cell Biology, Federal University of Paraná, Curitiba, Brazil; ^4^Institute of Tropical Medicine, University of Tübingen, Tübingen, Germany

**Keywords:** Chagas disease, autoimmunity, autoantibodies, chronic Chagas disease, mimicry, bystander activation, complement system

## Abstract

Chagas disease (CD), a neglected tropical disease caused by the protozoan *Trypanosoma cruzi*, affects around six million individuals in Latin America. Currently, CD occurs worldwide, becoming a significant public health concern due to its silent aspect and high morbimortality rate. *T. cruzi* presents different escape strategies which allow its evasion from the host immune system, enabling its persistence and the establishment of chronic infection which leads to the development of chronic Chagas cardiomyopathy (CCC). The potent immune stimuli generated by *T. cruzi* persistence may result in tissue damage and inflammatory response. In addition, molecular mimicry between parasites molecules and host proteins may result in cross-reaction with self-molecules and consequently in autoimmune features including autoantibodies and autoreactive cells. Although controversial, there is evidence demonstrating a role for autoimmunity in the clinical progression of CCC. Nevertheless, the exact mechanism underlying the generation of an autoimmune response in human CD progression is unknown. In this review, we summarize the recent findings and hypotheses related to the autoimmune mechanisms involved in the development and progression of CCC.

## Introduction

Chagas disease (CD) is a neglected tropical disease caused by the protozoan parasite *Trypanosoma cruzi*, which affects around six million individuals in Latin America (1). CD is increasing as a health threat in countries of Europe, the United States, Canada, Japan, and Australia, where blood transfusion, organ transplantation, and vertical transmission seem to be the main transmission routes (1). According to the World Health Organization, the estimated incidence of CD in the Americas is 30,000, followed by 14,000 deaths and 8,000 infected newborn per year (2).

**Figure 1:**
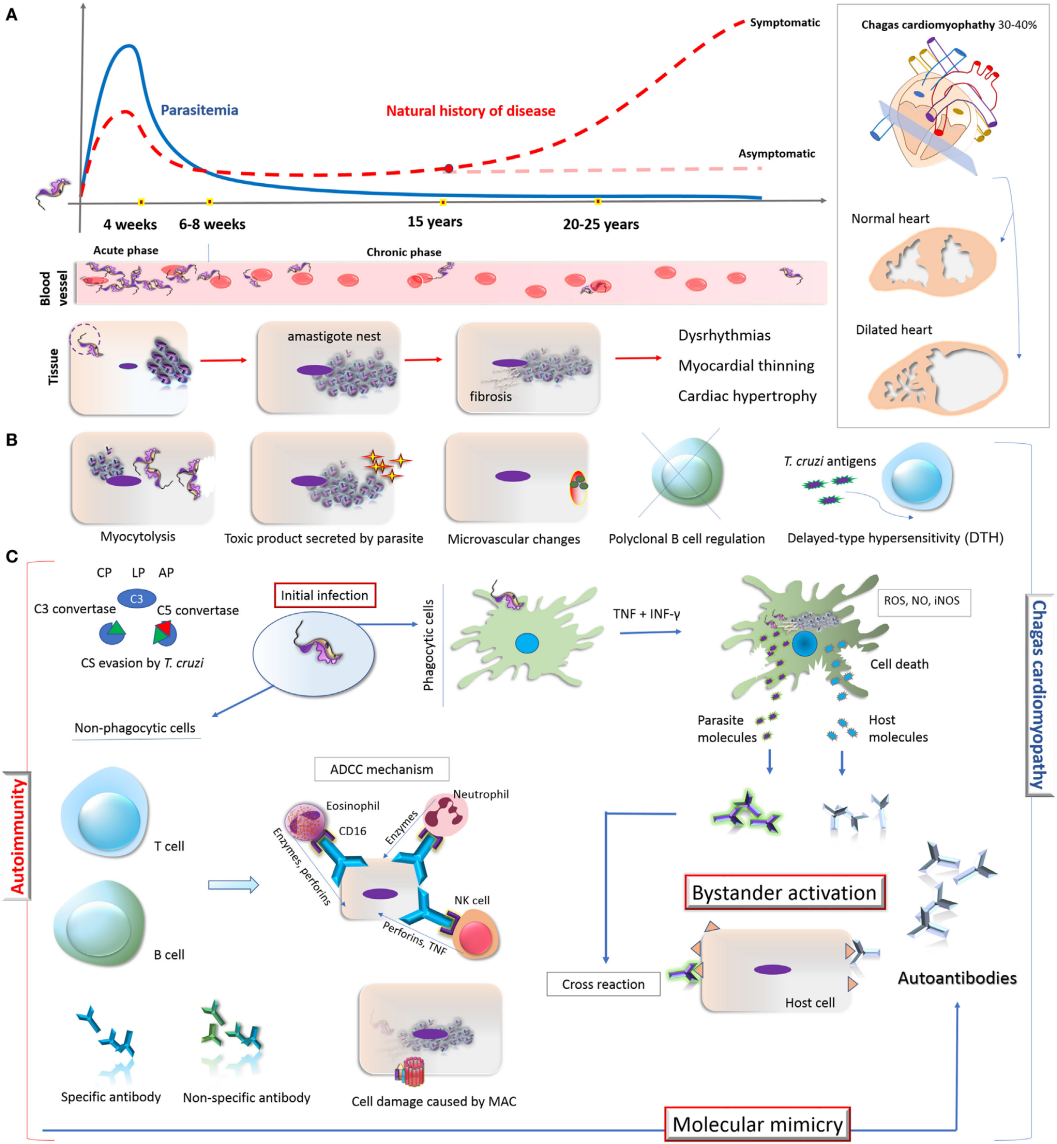
Overview on the natural history of CD, development of cardiomyopathy and its autoimmunity pathophysiological mechanisms. **(A)** Natural history of CD: the acute phase of *Trypanosoma cruzi* infection is oligosymptomatic and characterized by high parasitemia, which starts to decrease after 4 weeks. During the chronic phase (6–8 weeks), the parasitemia remains low and some patients (30–40%) might develop Chagas-related symptoms, especially cardiomyopathy. The parasite invades and differentiates in cardiomyocytes, leading to a fibrosis condition and consequently dysrhythmia, myocardial thinning, and cardiac hypertrophy. **(B)** Direct mechanisms associated with the cardiomyocyte damage: myocytolysis (cell lysis after amastigote differentiate into trypomastigote); toxic molecules produced by the parasite; microvascular changes induced by the parasite (cardiac hypoperfusion); disruption of immune regulation mechanisms in B cell (represented by X); constant presence of *T. cruzi* antigens triggers T cell-mediated damage and DTH process; autoimmunity (represented by the antibodies in the right). **(C)** Autoimmunity pathways in chronic CD: *T. cruzi* presents different escape strategies which enable its evasion from CS activation, allowing its entry in phagocytes, persistence, and the establishment of chronic infection which lead to the development of CCC. The potent immune stimuli generated by *T. cruzi* persistence (here represented by TNF, IFN-γ, ROS, NO, iNOS production by phagocytic cell) may result in tissue damage and inflammatory response through bystander activation and molecular mimicry. Bystander activation is caused by the exposure of both host and parasite intracellular proteins resulting in potent immune stimuli due to the release of self-antigens that induces the production of autoantibodies. Molecular mimicry occurs when there are structural similarities between *T. cruzi*-specific molecule and host-molecule, triggering T-cell activation. Specific antibodies from B cells can participate in ADCC mechanism on target cells. Neutrophil, eosinophil, and NK cell interact with these antibodies *via* CD16 (Fc receptor) and release lytic molecules like enzymes, perforins, or TNF on the target cells, independent of the CS. Moreover, CS activation and constant evasion strategies from *T. cruzi* could damage the host tissues through MAC formation. Abbreviations: CD, Chagas disease; DTH, delayed-type hypersensitivity; CCC, chronic Chagas cardiomyopathy; CP, classical pathway; LP, lectin pathway; AP, alternative pathway; CS, complement system; TNF, tumor necrosis factor; IFN-γ, interferon; ROS, reactive oxygen species; NO, nitric oxide; iNOS, inducible nitric oxide synthase; ADCC, antibody-dependent cell-mediated cytotoxicity; MAC, membrane attack complex; NK, natural killer cell.

Chagas disease is a life-threatening and persistent illness, having both acute and chronic phases (3). During the acute phase, which develops within a short time (4–8 weeks) following the infection, the parasite burden is controlled by the acute inflammatory response (4, 5). In order to establish a life-long infection, it is known that *T. cruzi* evades host immune response and, with this, some patients will remain asymptomatic and with low levels of intracellular parasites (6). The long-term proliferation and persistence of these parasites in the tissue leads to the establishment of the chronic phase of CD (7). Nearly 30–40% of chronically infected patients evolve from asymptomatic condition to symptomatic forms, including cardiac, digestive, or cardiodigestive (Figure 1A) (8).

There is a large variability in the outcome of *T. cruzi* infection, which is possibly due to different pathogenic mechanisms. However, the real contribution of the immunogenetic pattern of the human host, parasite diversity, and persistence, among others that could determine the clinical progression from asymptomatic to symptomatic CD forms remain enigmatic (9–14). In these circumstances, the parasite evasion of both humoral and cellular immune responses may lead to the success of *T. cruzi* infection and development of chronic CD (6, 9, 15–19).

Despite the contribution of the parasite persistence and the host genetics to the clinical progression of CD, it is known that immune reactivity against cardiac antigens (e.g., cardiac myosin) can occur during the infection in some patients (20, 21), where parasite-induced damage may lead to molecular mimicry between parasite/host proteins epitopes, thereby generating a potent immune stimuli (21). This may exceed the threshold of immune activation acceptable for self-tolerance, resulting in cross-reaction with self-molecules and, eventually, host tissue damage (6, 8, 21–25). Although the development of autoantibodies in CD has been demonstrated in several studies (17, 19, 22, 24, 26–34), its role in the clinical development of the disease has not been clarified. This review aims to address some of the possible mechanisms of autoimmunity involved in CD.

## From Infection to Immune Responses Evasion: What is the Consequence of Parasite Persistence?

*Trypanosoma cruzi* can be transmitted by vectors (bugs from *Triatominae* subfamily) as well as blood transfusions, organ transplantation, ingestion of food contaminated with the parasite, vertical transmission, among others (35). Through a process called adhesion and recognition, the parasite forms a stable bond with cell surface molecules that serve as adhesion anchors to the cell for invasion (16, 36). In vectorial transmission, the invasion of host cells occurs by metacyclic trypomastigotes, the infective stage of the parasite. After cell invasion, the trypomastigotes differentiate into amastigotes and replicate in the cytosol, where they differentiate into trypomastigotes and, with the rupture of the cell, these reach the bloodstream, spreading the infection to other tissues. Infective *T. cruzi* metacyclic trypomastigotes have the ability to invade any mammalian cell (7, 37, 38).

Both humoral and cellular immune responses are essential for parasite control (39). In this context, the host response uses several strategies to eliminate the parasite including complement activation (15, 40, 41), opsonization (42), production of specific antibodies (43–45), and antibody-dependent cellular cytotoxicity (46, 47). The complement is part of the innate immunity acting in the first line of host defense against pathogens (48). It comprises more than 35 proteins and can be activated by three pathways: lectin, classical, and alternative (15). As soon as the trypomastigotes reach the host bloodstream, lectin pathway (LP) and alternative pathway (AP) are activated since both pathways do not depend on specific antibody responses (49). Collectins and ficolins recognize and bind to glycosylated and acetylated molecules on the surface of *T. cruzi* trypomastigotes activating the LP and the AP is spontaneously activated by hydrolysis of C3 (12, 14, 15). As the infection progresses, the host can mount a specific antibody response against *T. cruzi* that will lead to interaction with C1 complex composed of one molecule of C1q and two molecules each of C1r and C1s—activating the classical pathway (CP) (49). Once activated, proteases from both LP and CP cleave C2 and C4, generating a C3 convertase (C4b2a) which cleaves the central complement component C3 in C3a and C3b. This last fragment binds to C3 convertase forming C5 convertase, which cleaves C5 in C5a and C5b. The fragment C5b binds to C6, C7, C8 and 12–18 copies of C9 and, as a final product of complement activation, the membrane attack complex (MAC) is formed on the target cell (such as epimastigotes), promoting its lysis (50).

*Trypanosoma cruzi* utilizes its surface proteins (such as *T. cruzi* calreticulin, trypomastigote decay-accelerating factor, *T. cruzi* complement regulatory protein—Gp160, *T. cruzi* complement C2 receptor inhibitor trispanning, and *T. cruzi* complement regulatory gp58/068) to circumvent complement-mediated lysis and opsonization (15, 51, 52). These proteins disturb the attachment of initial molecules from complement pathways, thereby inhibiting the C3 convertase formation, which is a crucial step in the activation of all three pathways and generation of complement-mediated effects (49, 53).

Metacyclic trypomastigote forms of *T. cruzi* not only involve the expression of regulatory molecules on parasite’s surface but they also induce membrane-derived vesicles (microvesicles) from host cells, which affects the formation and activation of C3 convertase (C4b2a), resulting in the inhibition of complement activation, increased parasite survival, and eukaryotic cell invasion (54). Moreover, microvesicles derived from both host cells and *T. cruzi* can fuse, thereby increasing host cell invasion and parasite dissemination (55). Both mechanisms interfere also in the activation of complement (49, 54). Incomplete parasite clearance may lead to immune reactivity that could elicit tissue damage leading to the exposure of neoepitopes and stimulus for autoantibody production (56), triggering mechanisms involved in autoimmunity in chronic CD (8). These mechanisms are demonstrated in Figure 1.

## Autoimmunity in CD

The pathogenesis of symptomatic chronic CD is not yet completely understood. Some hypotheses are based on the direct response of the immune system against infected tissues (8, 57, 58). According to these hypotheses, an efficient immune response could result in the substantial reduction in the number of parasites with less tissue damage and lack of clinical manifestations in asymptomatic patients (58). On the other hand, an inefficient immune response would favor parasite persistence in the tissues with consequent injury and fibrosis (59, 60). In addition, the autoimmunity hypothesis suggests that cardiac damage, triggered by parasite persistence, would lead to an exacerbation of the immune response and disruption in self-tolerance, resulting in immune reaction against self-molecules (58). In this case, the autoimmune response would possibly be the reason for the late damage observed in chronic CD (8). In fact, the first evidence of autoimmunity in CD was presented by Cossio and collaborators in 1974 (61), who reported antibodies in sera of chagasic patients that reacted with endocardium, interstitium, and heart blood vessels but were absent in healthy individuals and in patients with non-chagasic cardiomyopathy. The involvement of autoimmunity in the pathogenesis of chronic Chagas cardiomyopathy (CCC) has been extensively studied (18, 24, 25, 62–64), although questions concerning its exact role remain unanswered (58). It is worth mentioning that, in addition to autoimmunity, other mechanisms may contribute to the development of CCC, such as myocytolysis, secretion of toxic molecules by the parasite, microvascular changes induced by the parasite (cardiac hypoperfusion), disruption of immune regulation mechanisms in B cell and T cell-mediated delayed-type hypersensitivity (8, 57, 58, 65, 66) (Figure 1B). In general terms, the autoimmunity in CD has been considered one of the key mechanisms to explain the tissue damage observed in the chronic phase, even in the absence of the parasite in the affected tissues (Figure 1C).

## How Does Autoimmunity Lead to Autoantibody Development?

Two main mechanisms support the autoimmunity hypothesis in CCC: bystander activation and molecular mimicry (Figure 1C). The first involves the exposure of intracellular proteins after parasite-induced damage, resulting in the release of self-antigens in an inflammatory environment. In addition, the constant presence of parasite antigens can trigger responses mediated by CD4^+^ and/or CD8^+^ T cells, which may be responsible for injuring infected or neighboring tissue cells (59, 67–69). This potent immune stimulus may overcome the threshold of self-tolerance and trigger the production of autoantibodies targeted to multiple antigens (58). In case of molecular mimicry, sequence similarities between foreign and self-peptides result in the cross-activation of autoreactive T or B cells to the host peptides (62, 63, 67). Cumulative evidence of cross-reactivity between *T. cruzi* and human antigens as well as of autoantibodies affecting structures and functions of the heart muscle have been reported (18, 25, 58).

Several mechanisms involved in the pathogenesis of CD suggest that the autoimmune aggression in the muscle fiber is due to antigenic mimicry to *T. cruzi* and host molecules. The similarity of antigenic epitopes of the parasite and host tissue leads to cross-reaction and production of autoreactive antibodies. In fact, molecular mimicry is considered the most significant mechanism of autoimmunity in CCC, being a key pathogenic event in disease manifestation. The demonstration that CD4^+^ T cells from mice with chronic Chagas myocarditis were able to transfer the cardiac damage to healthy mice corroborates this hypothesis (21). In addition, passive transfer of serum and/or antibodies from chagasic patients presenting complex cardiac arrhythmias were able to induce disturbances in the electrogenesis and conduction of adult rabbit hearts, confirming the pathogenicity of CD autoantibodies (70, 71). Moreover, *T. cruzi* antigens that mimic human host antigens evidence the connection between parasite persistence and autoimmunity (18). The cross-reactivity between host molecules and *T. cruzi* antigens is listed in Table 1.

**Table 1:** Cross-reactivity and human autoantibodies described in Chagas disease.

	*Trypanosoma cruzi* antigens	Autoantibody	Target human epitope	Reference
Nervous system	Sulfated glycolipid	Anti-neuron	Neurons of the central and peripheral nervous system	(26)
		Anti-sciatic nerve	Sciatic nerve components	(72)
	
	Cytoplasmic ribosome	Anti UsnRNPs	Small nuclear ribonucleoproteins (UsnRNPs)	(28)
	
	FL-160 surface protein	Anti-FL-160	Neuronal protein 48 kDa	(73)
	
	Microtubule-associated protein (MAP)	Anti-MAP like protein	MAP of brain	(74)

Heart	Glycosphingolipids	Anti-neutral glycosphingolipids	Glycosphingolipids from heart muscle cells	(75)
	
	Ribosomal P0 and P2β (TcP2β) proteins	Anti-β1 adrenoreceptor	C-terminal region of the ribosomal P proteins similar to the second extracellular loop of β1 adrenoreceptor	(19, 76)
		Anti-β1 adrenoreceptors	Myocardial β1 adrenoreceptor	(77)
	
	B13 protein	Anti-cardiac myosin heavy chain	Cardiac myosin heavy chain	(20)
	
	Cruzipain	Anti-mAChR	Heart cardiac muscarinic acetylcholine receptor (mAChR)	(17)
			Second extracellular loop of the human heart mAChR	(34, 78)
			Third extracellular loop of the human mAChRs	(33)
	
	Cross-reacting antigen (SRA) on striated muscle	Anti-SRA	SRA on the sarcolemma of cardiac myofibers	(79)
	
	Microsomal fraction (Mc)	Anti-Mc antibodies	Skeletal and heart muscle	(80, 81)

Immune response	55 kDa membrane protein	Anti-B lymphocytes p28	28 kDa lymphocyte membrane protein	(82)
	
	Lectin domain of shed acute-phase antigen (SAPA)	Anti-Galectin-1	Galectin-1	(32)
	
	SAPA	Anti-Cha	Peptides R3 from human the autoantigen Cha	(83)

Others	23 kDa ribosomal protein	Anti-ribossomal P proteins	23 kDa ribosomal protein	(29, 84)
	
	P2β (TcP2β) protein	Anti-β2 adrenoreceptors	Spleen cell β2 adrenoceptors	(77)

In the course of CD, the most probable routes to the development of autoantibodies include (i) the exposure of intracellular proteins leading to bystander activation mechanism, (ii) molecular mimicry, and (iii) the polyclonal B cell activation (67) (Figure 1). Actually, antibodies against self-antigens such as actin, myosin, myoglobin, DNA, tubulin (85), desmin, and myosin from cardiac muscle (86) were found in animal models of *T. cruzi* infection. In patients with CD, autoantibodies targeting β1-adrenergic receptors (76) and muscarinic acetylcholine receptors (M2) were found associated with the development of cardiomyopathy (33, 78). Moreover, an association of anti-muscarinic receptors antibodies with ventricular electrical instability and sudden death in patients with CCC has been reported (87, 88). In addition, specific antibodies against cardiac myosin concomitantly with a robust autoreactive T-cell reaction increasing the production of different autoantibodies have been described (27, 89–91). Some of these autoantibodies are listed in Table 1.

The cell injury seen in CCC may be associated with antibody-dependent cell-mediated cytotoxicity (ADCC) since it has been shown that infected mice neutrophil, eosinophil, and natural killer cells interact *via* Fc receptor with antibodies, releasing lytic molecules such as perforins and tumor necrosis factor, leading to cytotoxic effect of target cells (92–94).

Furthermore, complement activation and formation of MAC on host cell surface may be involved in the cell injury process present in CCC. During the chronic phase of CD, which is known to be associated with ongoing inflammation, complement becomes activated, resulting in the assembly of MAC on endothelial and cardiomyocyte cells and causing tissue damage (95). This event could explain, in part, the active myocarditis and the fibrosis observed in myocardial lesions seen in some patients with chronic CD. The formation of MAC on cardiomyocytes of chagasic patients suggests that cell damage would favor exposure of intracellular molecules and the development of autoantibodies contributing to the autoimmunity process (96) (Figure 1C). Although the presence of autoantibodies might represent a factor involved in cell damage in the chronic phase of CD, the real effect of autoimmunity in the clinical development of the disease is still unknown.

Thus, understanding the autoimmunity hypothesis in CCC development may guide new strategies for the treatment of chronic CD. Nevertheless, one may consider that therapies modulating the immune response are complex and may be a double-edged sword causing side effects since the abrogation of molecules from the immune system in experimental *T. cruzi* infection have shown to increase parasitemia (97).

## Final Considerations

The autoimmune hypothesis in the pathogenesis of CD is a topic of controversial debate, and several studies have demonstrated the involvement of more than one plausible mechanism that could contribute to the tissue damage observed in the chronic phase of the disease. Thus, the presence of the parasite within the tissues could stimulate in a continuous way both humoral and cellular responses activating multiple pathways, such as molecular mimicry and autoantibodies formation, bystander activation, ADCC, and complement activation, contributing to tissue damage and progression to symptomatic forms, including chagasic cardiomyopathy. All the information gathered in this review contributes to highlight points of possible interventions for future development of strategies regarding neutralization, blocking or immunoadsorption of autoantibodies, as well as complement inhibition. Thus, a better understanding of the host immune response during *T. cruzi* infection and CD progression is a key element to the development of effective vaccines and immunotherapy.

## Author Contributions

EB, KCFL, ZO, LB, LHG, TLS, and IJMR participated in the design and writing of the manuscript. TLS and IJMR participated in the design, coordination, and manuscript writing. KCFL developed the figure graphic design.

## Conflict of Interest Statement

The authors declare that the research was conducted in the absence of any personal, professional, commercial, or financial relationships that could be construed as a potential conflict of interest.
